# Organoborane coupling reactions (Suzuki coupling)

**Published:** 2004-08-01

**Authors:** Akira Suzuki

**Affiliations:** Professor Emeritus, Hokkaido University

**Keywords:** Pd-catalyst, cross-coupling reaction, organoboron compounds, synthesis of conjugated alkadienes and alkenynes, biaryl synthesis

## Abstract

The palladium-catalyzed cross-coupling reaction between different types of organoboron compounds with sp^2^-, sp^3^-, and sp-hybridized carbon-boron compounds and various organic electrophiles in the presence of base provides a powerful and useful synthetic methodology for the formation of carbon-carbon bonds. The coupling reaction offers several advantages:

1. Availability of reactants
2. Mild reaction conditions
3. Water stability
4. Easy use of the reaction both in aqueous and heterogeneous conditions
5. Tolerance of a broad range of functional groups
6. High regio- and stereoselectivity
7. Insignificant effect toward steric hindrance
8. Use of very small amounts of catalysts
9. Utilization as one-pot synthesis
10. Non-toxic reaction

## Introduction

Carbon-carbon bond formation reactions are important processes in chemistry, because they provide key steps in the building of more complex molecules from simple precursors. Over the last several decades, reactions for carbon-carbon bond formation between molecules with saturated sp^3^ carbon atoms have been developed. There were no simple and general methods, however, for the reactions between unsaturated species such as vinyl, aryl, and alkynyl moieties until the discovery and development of metal-mediated cross-coupling reactions, starting in the 1970s. In 1972, Kumada1) and Corriu2) independently reported that the reaction of Grignard reagents with alkenyl and aryl halides can be catalyzed by Ni(II) complex. Thereafter, several coupling reactions of Grignard reagents and other organometallic compounds were reported by palladium catalysts. The recent progress of these cross-coupling reactions has been summarized in book form.3)

On the other hand, organoboron compounds have many advantages, compared to other organometallic derivatives, i.e. ready availability and stable character, etc. Consequently, many chemists attempted to find such cross-coupling reactions using organoboron compounds, but there were no successful reports on the organoborane coupling reaction.

In the mid 1970s, we were attempting to find a stereo- and regioselective synthesis for conjugated alkadienes, because these compounds have interesting biological activities and are of great importance in organic chemistry. A number of methods for the preparation of conjugated dienes and polyenes were developed utilizing organometallic compounds. Although some of such procedures proved to be successful, the scope of these reactions was limited either by the nature of the organometallic reagent involved or by the procedure employed. The most promising procedure for preparing conjugated dienes or enynes in a selective manner was considered to be the direct cross-coupling reaction of stereodefined haloalkenes or haloalkynes with stereodefined alkenylboron compounds. In spite of efforts by many chemists to find such general cross-coupling reactions using alkenyl boranes, there were no successful reports on the synthesis of conjugated unsaturated compounds at the time we commenced this study. Thereafter, we discovered fortunately that such an organoborane coupling reaction proceeds smoothly in the presence of a catalytic amount of palladium complex and base to provide the expected coupled products regio- and stereoselectively in high yields.

In this article, the cross-coupling reactions of organoboron compounds are described briefly in the order of 1-alkenyl, aryl, 1-alkyl-, and 1-alkynylboron derivatives, which are the order of discovery of the reactions.

## Coupling reactions of (sp^2^)C-B compounds. Vinylic boron compounds

### Reaction with vinylic halides. Synthesis of conjugated alkadienes

As mentioned in Introduction, cross-coupling reactions between vinyl boranes and vinyl halides were reported not to proceed smoothly in the only presence of palladium catalysts. During the initial sage of our exploration, we postulated that the reason for the drawback in the coupling is caused by the following aspects of the mechanism. The common mechanism of transitionmetal catalyzed coupling reactions of organometallic compounds with organic halides involves sequential (a) oxidative addition, (b) transmetalation, and (c) reductive elimination.3) One of the major reasons why 1-alkenylboranes cannot react with 1-alkenyl or 1-alkynyl halides appeared to be step (b). The transmetalation process between RMX (M = transition metal, X = halogen) and organoboranes does not occur readily because of the weak carbanion character of the organic groups in the organoboranes. To overcome this difficulty, we anticipated the use of tetracoordinate organoboron compounds, instead of tricoordinate organoboron derivatives. According to the study by Gropen and Haaland,4) the methyl group in tetramethylborate was observed to be 5.5 times more electronegative than the methyl group in trimethylborane. Such behavior was also expected for the reaction of triorganoboranes in the presence of base. Thus, we found that the rection of vinyl boron compounds with vinyl halides proceeds smoothly in the presence of a base and a catalytic amount of a palladium complex to provide the expected conjugated alkadienes and alkenynes stereo- and regioselectively in excellent yields (Table I).

Although the coupling reaction of (E)-1-alkenylboranes, readily obtained via the hydroboration of appropriate alkynes with disiamylborane or dicyclohexylborane, proceeds readily with (E)- and (Z)-1-alkenyl bromides and iodides to give the corresponding dienes readily (Table II), (Z)-1-alkenylboranes, prepared by hydroboration of 1-haloalkynes followed by the reaction with t-butyllithium, gave product yields, near 50% (Table III).

Fortunately, it became apparent that high yields and stereoselectivities could be achieved by coupling (Z)-1-alkenyl halides with (Z)-1-alkenyldialkoxyboranes, instead of disiamyl- and dicyclohexylborane derivatives, as shown in Table III.5) Consequently, the cross-coupling reaction of 1-alkenylboranes with 1-alkenyl halides can be achieved for syntheses of all conjugated alkadienes. The reaction has been applied to syntheses of many natural and unnatural compounds which have conjugated alkadiene structures.6) For example, enantioselective total synthesis of an antitumor antibiotic, vicenistatin has been achieved by Suzuki coupling as a key reaction (Eq. 1).7)

(Eq. 1)
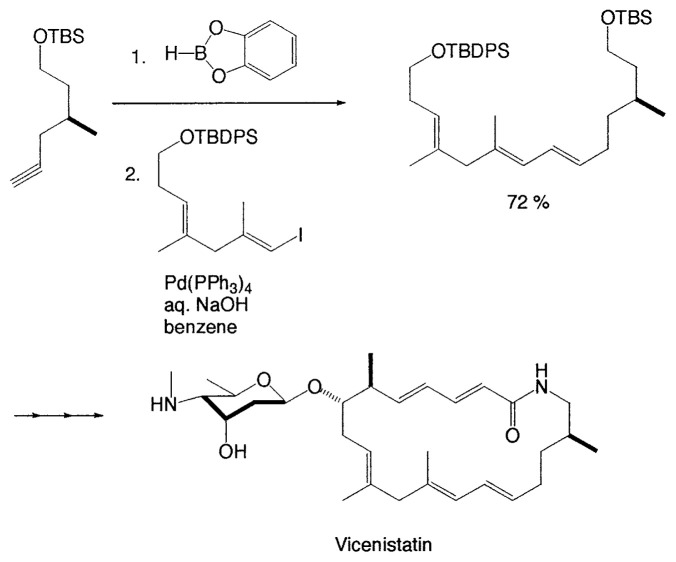


Rutamycins are cytotoxic and have potent antifungal activity. Total synthesis of rutamycin B has been carried out by the intramolecular Suzuki coupling reaction (Eq. 2).8)

In improved synthesis of (14*E*)- and (14*Z*)-dehydrocrepenynate, key intermediates in plant and fungal polyacetylene biosynthesis, the Suzuki coupling has been applied (Eq. 3).9)

Among many synthetic applications of Suzuki coupling reaction, the total synthesis of palytoxin, a complex and toxic natural product is an epoch-making contribution. 10)

(Eq. 2)
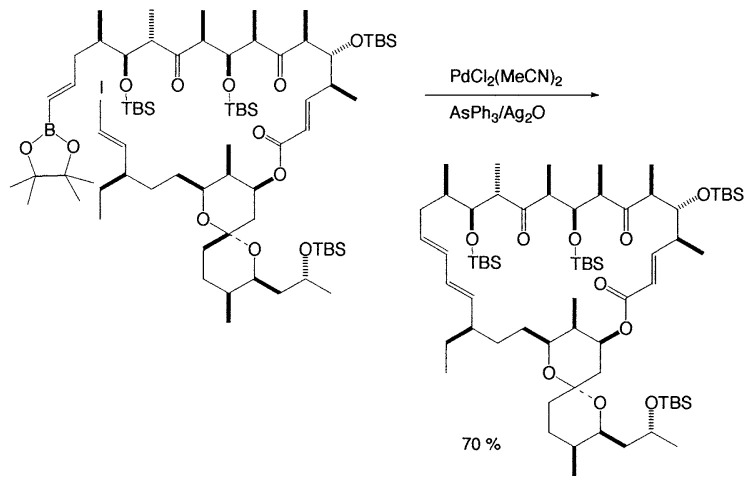


(Eq. 3)
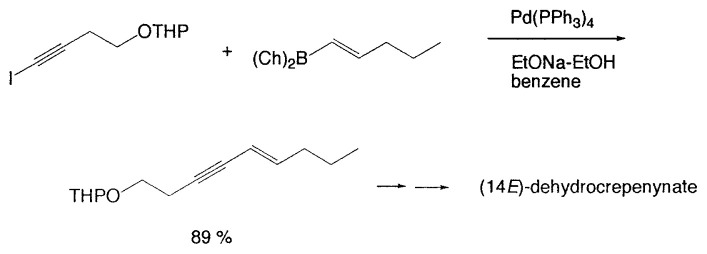


### Mechanism of the vinyl-vinyl cross-coupling

The principal features of the cross-coupling reaction are as follows: (a) Small catalytic amounts of palladium complexes (1–3 mol%) are required to obtain the coupled products. (b) The coupling reactions are highly regioand stereoselective and take place while retaining the original configurations of both the starting alkenylboranes and the haloalkenes. The isomeric purity of the products generally exceeds 97%. (c) A base is required to carry out a successful coupling. In the initial stage of the study, as mentioned previously, we considered that tetracoordinate organoboron compounds facilitate the transfer of organic groups from the boron to the palladium complex in the transmetalation step. In order to check this possibility, the reaction of lithium (1-hexenyl) methyldisiamylborate was examined as shown in Eq. 4. The coupled product, however, was obtained only in 9%. On the other hand, it was found that (trichlorovinyl)palladium(II) complexes **6** and **9** both prepared as pure solids, reacted with vinylborane **7** to give diene **8**, as depicted in Eqs. 5 and 6. In the case of **6**, no reaction occurs without a base, whereas the coupling reaction proceeds smoothly in the presence of a base to give the coupled product in 89% yield. The intermediate **9** readily reacts with **7** without a base to provide the same product **8** in almost quantitative yield after 1 h.

(Eq. 4)
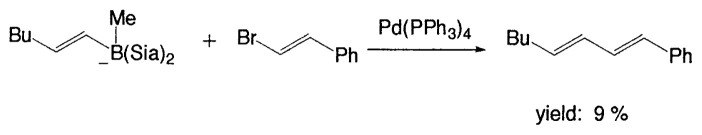


(Eq. 5)
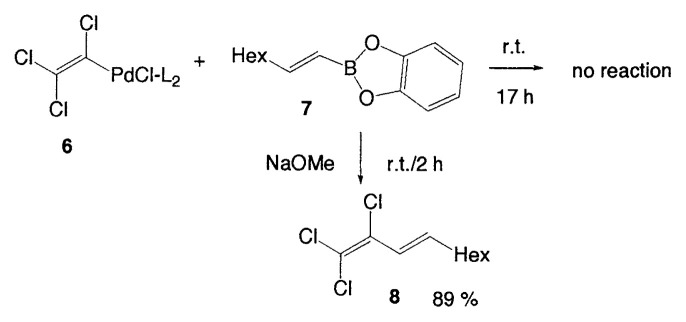


(Eq. 6)
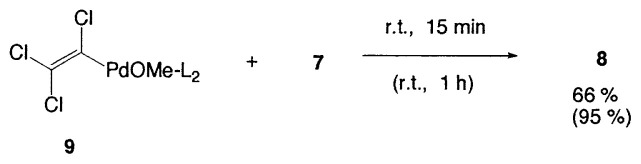


Consequently, such evidence suggests that vinylalkoxy-palladium( II) compounds such as **9** were necessary intermediates in these cross-coupling reactions. Accordingly it is considered that the reaction proceeds through the catalytic cycle as shown in Fig. 1.11)

### Reaction with aryl halides

As described in the previous section, it was discovered that vinylic boron compounds readily react with vinylic halides to give coupled products. We next attempted to examine the reaction of 1-alkenylboranes with haloarenes which have also sp^2^ hybridized carbon-halogen bonds, and found that the reaction takes place smoothly. Representative results are exhibited in Table IV.

This reaction has one more advantage that only one product **11** (head-to-head coupled product) is formed. Additional coupling reactions of vinyl boranes are shown in Table V. Aromatic bromides and iodides easily react with vinylic boron compounds, but aromatic chlorides do not participated for the coupling, except reactive chlorides, such as allylic and benzylic derivatives. Heteroaromatic halides can be used as coupling partners. Ortho-substituents on benzene ring do not give difficulty. Thus, the cross-coupling reaction is used for the synthesis of benzo-fused heteroaromatic compounds (Eq. 7).12)

(Eq. 7)
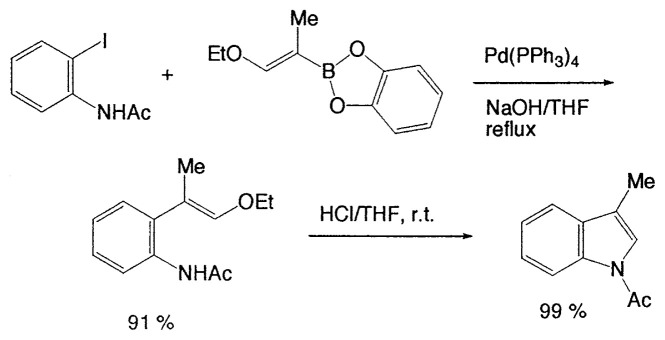


## Aromatic boron compounds

### Reaction with aromatic halides. Synthesis of biaryls

The coupling of aryl halides with copper at drastic high temperature is called the Ullmann reaction, which is of broad scope and has been used to prepare many symmetrical biaryls. However, when a mixture of two different aryl halides is used, there are three possible products. Consequently, the development of a selective and general synthesis of all kinds of biaryls has been desired.

The first method to prepare biaryls by the cross-coupling of arylboranes with haloarenes was observed in 1981 (Eq. 8).13) The reaction proceeds even under heterogeneous conditions to give the corresponding coupled products selectively in high yields. After this discovery, various modifications have been made to the reaction conditions. As the bases, Na_2_CO_3_, NaHCO_3_, Tl_2_CO_3_, K_3_PO_4_, etc. are employed. In some cases, CsF or Bu_4_NF can be used instead of usual bases (Eq. 9).14) Phosphine-based palladium catalysts are generally employed since they are stable on prolonged heating; however, extremely high coupling reaction rate can be sometimes achieved by using palladium catalysts without a phosphine ligand such as Pd(OAc)_2_, [(η^3^-C_3_H_5_)PdCl]_2_, and Pd_2_(dba)_3_.

(Eq. 8)
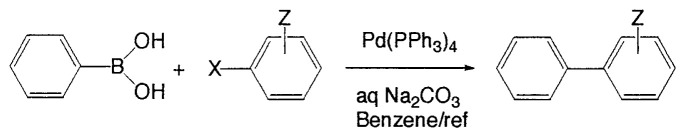


(Eq. 9)
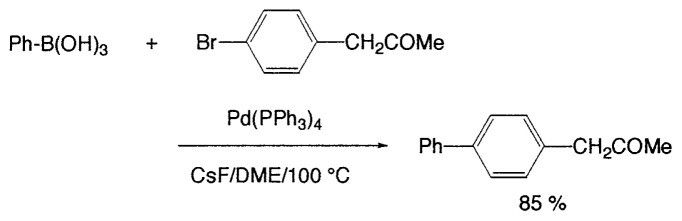


Carbon-carbon bond formation reactions employing organoboron compounds and organic electrophiles have been recently recognized as powerful tools for the construction of new organic compounds. Among such reactions, aromatic-aromatic (or heteroaromatic) couplings between aromatic boronic acids or esters and aromatic electrophiles providing symmetrical and unsymmetrical biaryls selectively in high yields have been used most frequently. The importance of biaryl units as components in many kinds of compounds, pharmaceuticals, herbicides, and natural products, as well as engineering materials, such as conducting polymers, molecular wires, and liquid crystals has attracted enormous interest from the chemical community. Such aromaticaromatic, aromatic-heteroaromatic, and heteroaromatic-heteroaromatic coupling reactions have been recently reviewed in detail.15)

### Coupling of arylboronic acid derivatives having highly steric hindrance or electron-withdrawing functionalities

Although steric hindrance of aryl halides is not a major factor for the formation of substituted biaryls, low yields are resulted when *ortho*-disubstituted arylboronic acids are used. For example, the reaction with mesitylboronic acid proceeds only slowly because of steric hindrance during the transmetalation to palladium(II) complex. The reaction of mesitylboronic acids with iodobenzene at 80 ˚C in the present of Pd(PPh_3_)_4_ and various bases has been reported.16) The results are summarized in Table VI.

Aqueous Na_2_CO_3_ in benzene or DME (dimethoxyethane) is not effective as a base for the coupling of mesitylboronic acid and the reaction is not completed even after 2 days. Although the side reactions such as homocoupling are negligibly small, the formation of mesitylene was observed by hydrolytic deboronation increasing with the reaction time. It is noteworthy that such hydrolytic deboronation is faster in benzene/H_2_O than the modified conditions using aqueous DME. On the other hand, the addition of stronger bases, e.g., aqueous NaOH or Ba(OH)_2_, both in benzene and DME exerts remarkable effect on acceleration of the rate of coupling. By using aqueous Ba(OH)_2_ in DME at 80 ˚C, mesitylboronic acid couples with iodobenzene within 4 h to give the corresponding biaryl in a quantitative yield. Some of such coupling reactions are depicted in Eqs. 10 and 11.

(Eq. 10)
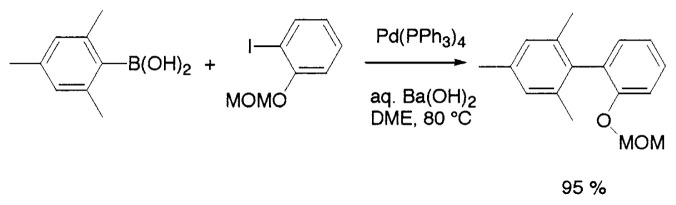


(Eq. 11)
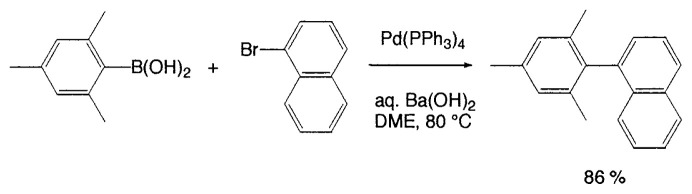


An alternative procedure, using the esters of boronic acids and anhydrous base has been developed for sterically hindered arylboronic acids, providing high yields. The coupling can be readily achieved by using trimethylene glycol ester of mesitylboronic acid and Cs_2_CO_3_ or K_3_PO_4_ in DMF at 100 ˚C to give a quantitative yield of the coupled products (Eq. 12).16)

(Eq. 12)
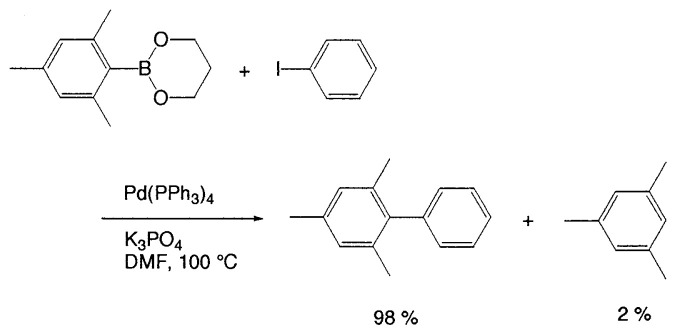


Even without sterically hindered substrates, the reaction under aqueous conditions is often undesirable because of competitive hydrolytic deboronation. Kinetic study17) into the reaction of substituted arylboronic acids showed that electron-withdrawing substituents accelerate the deboronation. Although there is no large effect between *meta* and *para* substituted phenylboronic acids, substituents at the *ortho* position may greatly increase the rate of deboronation. For example, the 2-formyl group on arylboronic acids is known to accelerate the rate of hydrolytic deboronation. 17) Indeed, the coupling of 2-formylphenylboronic acid with 2-iodotoluene at 80 ˚C using Na_2_CO_3_ in DME/H_2_O gives only a 54% yield of the corresponding biaryl, with accompanying benzaldehyde (39%). Aprotic conditions are desirable for such boronic acids sensitive to aqueous base. Thus, the trimethylene glycol ester of 2-formylphenylboronic acid readily couples with iodobenzene at 100 ˚C in DMF to give the coupled product in a yield of 89%, with less than 10% of benzaldehyde formation (Eq. 13).16)

(Eq. 13)
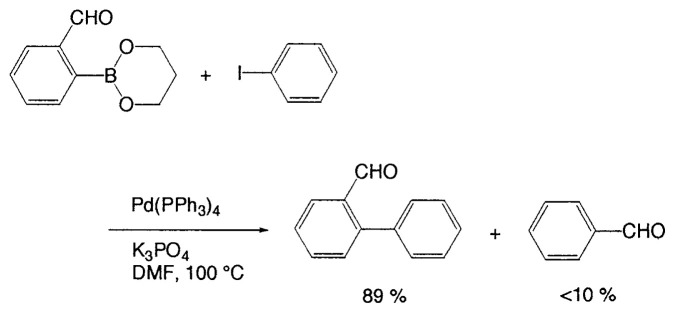


Recently, Buchwald *et al*. have reported interesting catalysts and ligands to prepare tetra-*ortho*-substituted unsymmetrical biaryls.18) Among biphenyl-based ligands, **14** gave an excellent result, whereas significant amounts of aryl bromide reduction were observed when the ligands **13** were used (Table VII).

### Coupling with aromatic chlorides

In aromatic-aromatic cross-coupling reactions, cheap and readily accessible aryl chlorides are particularly important as starting materials from an industrial viewpoint. Recently some of research groups, especially Fu’s group19) and Buchwald’s group20) have reported very efficient methods for aryl chloride reaction. For example, Fu and his coworkers19) have observed that the use of Pd_2_(dba)_3_/P^t^Bu_3_ as a catalyst and ligand for a wide range of aryl and vinyl halides, including chlorides, undergo Suzuki cross-coupling with arylboronic acids in very good yield, typically at room temperature (Table VIII). Furthermore, these catalysts display novel reactivity patterns, such as the selective coupling by Pd_2_(dba)_3_/PCy_3_/KF of a sterically hindered aromatic chloride (Eq. 14).

Despite the good yields in many Suzuki reactions of chloroarenes, generally, comparatively large amounts of catalyst are required (1–3 mol%). Beller *et al*. reported a new catalyst system, with which they achieved the coupling of nonactivated and deactivated aryl chlorides highly efficiently in good yields with generally only 0.005 mol% palladium and thus under industrially viable.21) For instance, as new efficient catalyst system, they used diadamantyl-*n*-butylphosphane (BuPAd_2_) as a ligand and found that it proved to be extremely reactive. One of typical examples is shown in Eq. 15.

(Eq. 14)
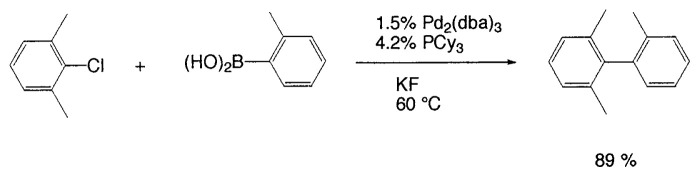


(Eq. 15)
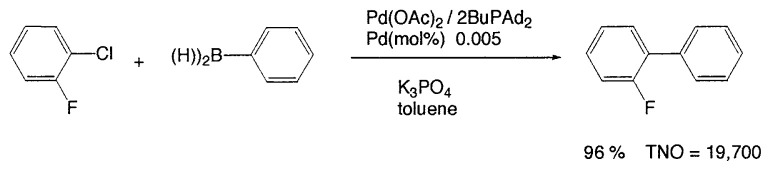


### Applications to synthesis of biaryls

The anti-HIV alkaloids, michellamine A **17** and B **18** have been synthesized. The tetraaryl skeleton of the michellamines was constructed by formation, first, of the inner (nonstereogenic) biaryl axis and subsequently of the two other (stereogenic) axes by using a double Suzuki-type cross-coupling reaction between the dinaphtalene ditriflate **15** and isoquinolineboronic acid **16** (Eq. 16).22)

(Eq. 16)
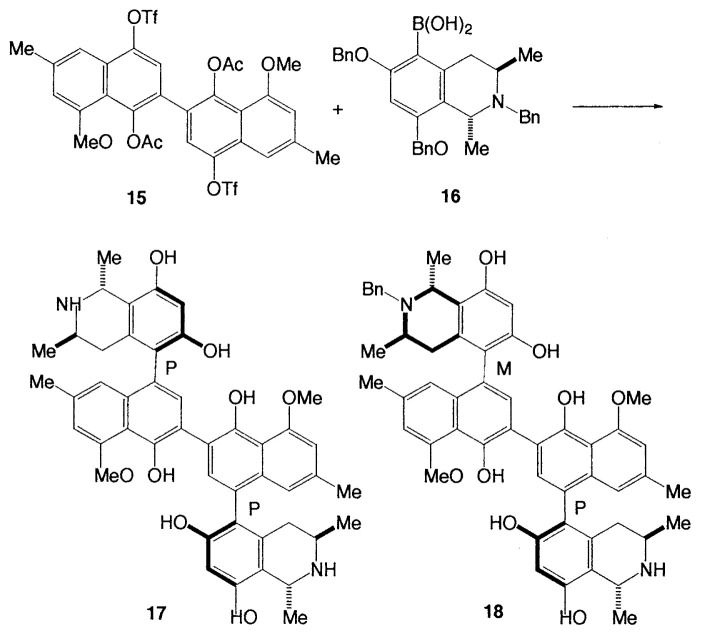


The discovery and development of penicillin and other antibacterial agents as drugs to fight infectious diseases were milestone victories of humankind over bacteria. While these agents saved millions of lives, they did not tame bacteria. On the contrary, this war led to the emergence of newer and more dangerous bacterial strains that responded against known antibacterial agents. Vancomycin is a member of the polycyclic glycopeptide class of antibiotics and has proved to be the last line of defense against drug-resistant bacteria. The daunting synthetic challenge posed by its structure is largely due to the strained nature of the 12-membered biaryl framework (AB ring system) and the two 16-membered biaryl ethers (COD and COE ring systems). Nicolaou and his group reported a Suzuki coupling approach to the AB-COD bicyclic system of vancomysin. 23) Suzuki coupling of the iodide **19** with **20** was facilitated by Pd(PPh_3_)_4_ catalyst and Na_2_CO_3_ to give a 1:1 mixture of the two atropisomers **21a** and **21b** in 80% combined yield (Eq. 17). The coupling of the parent boronic acid corresponding to **20** (without methyl groups) with iodide **19** led to a single compound. Thereafter, the total synthesis of the vancomycin aglycon has been reported by the same workers.24)

(Eq. 17)
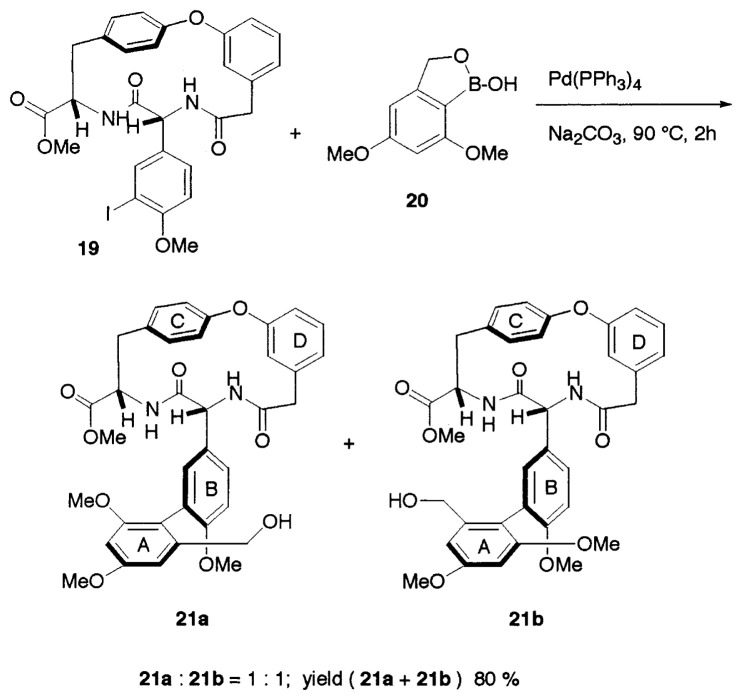


The novel class of tetrakis(phenothiazinylphenyl)-methane **23**, showing remarkably large Stokes shift and a reversible low oxidation potential, can be prepared in a good yield by Suzuki coupling of tetrakis(*p*-bromophenyl) methane **22** (Eq. 18).25)

(Eq. 18)
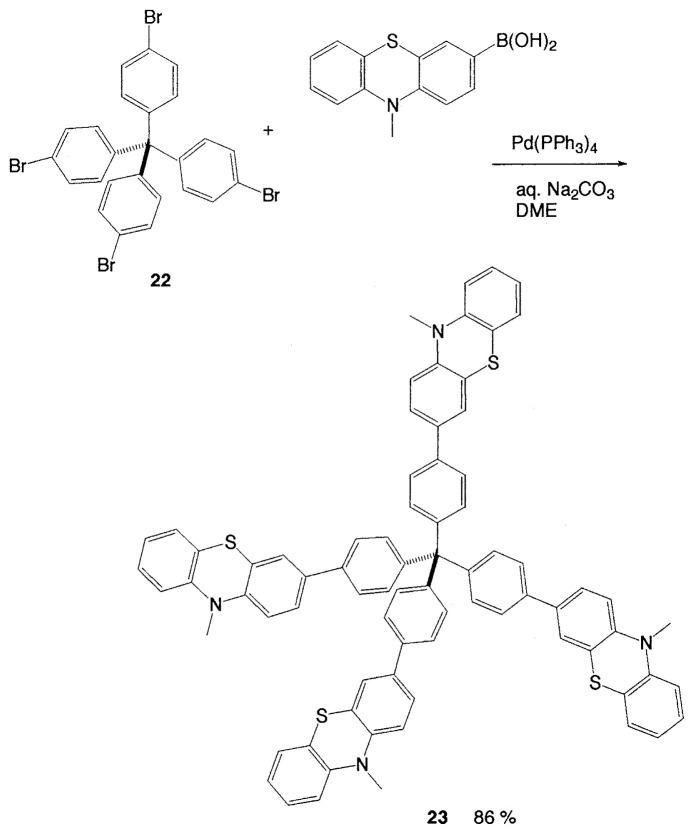


Oligothiophene functionalized 9,9-spirobifluorene derivatives have been synthesized by Suzuki coupling in high yields. The Negishi coupling reaction between oligothienylzinc chloride and various 9,9′-spirobifluorene bromides with Pd(PPh_3_)_4_ as catalyst successfully produce the desired compounds. However, the Negishi coupling provided low yields, compared to Suzuki coupling (Eq. 19).26)

(Eq. 19)
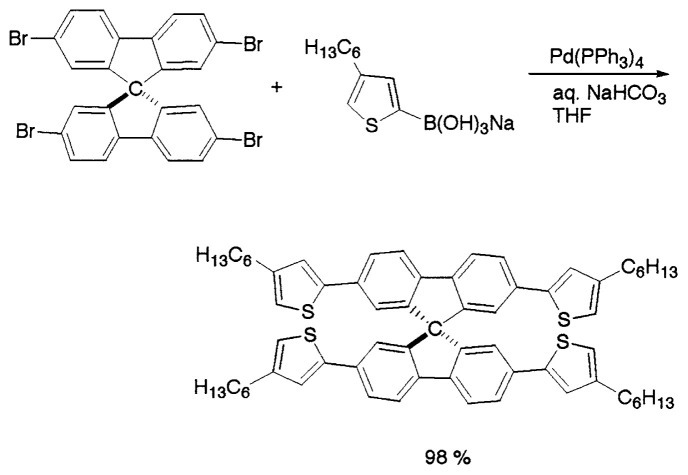


### Solid-phase synthesis (combinatorial methodology)

Solid-phase reactions play an important role in parallel synthesis and combinatorial chemistry, particularly in the area of medicinal chemistry, where their potential has emerged as a result of the possibility of automation. A considerable amount of attention has been focused on adapting and exploiting the advantage of solid-phase synthesis (SPS) for the production of libraries of such organic compounds. In this context, transition metal-promoted reactions serve as efficient methods because they proceed under mild conditions and are compatible with many functional groups. For instance, solid-phase Suzuki coupling has been largely developed mainly by the reaction of a resin-bound aryl halide with a solution-phase boronic acids.15) Recently, the viability of solid-supported boronic acids as reagents for Suzuki couplings was successfully demonstrated.27)

### Applications in polymer chemistry

**Fig. 2:**
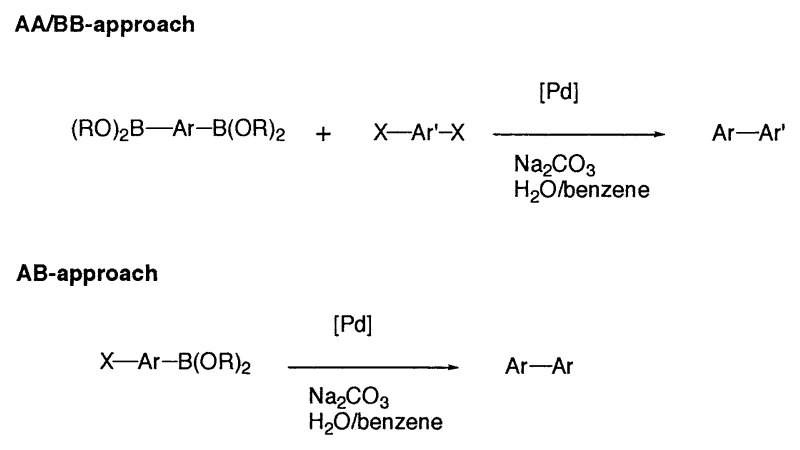
Graphical representation of Suzuki polycondensation

Aromatic, rigid-rod polymers play an important role in a number of diverse technologies including high-performance engineering materials, conducting polymers, and nonlinear optical materials. The Suzuki polycondensation (SPC) reaction of aryldiboronic acids and dihaloarenes for the synthesis of poly(*p*-phenylenes) was first reported by Schlüter.28a) SPC is a step-growth polymerization of bifunctional aromatic monomers to poly(arene)s and related polymers (Fig. 2).28b) The required functional groups, boronic acids or esters on the one side and bromide, iodide and so forth on the other, may be present in different monomers (AA/BB approach) or combined in the same monomer (AB approach).

The method was extensively applied to monodisperse aromatic dendrimers, water-soluble poly(*p-*phenylene), planar poly(*p*-phenylenes) fixed with the ketoimine bonds, poly(phenylenes) fused with poly-cyclic aromatics, and nonlinear optical materials.15) Here, one of such applications is shown (Eq. 20).29)

(Eq. 20)
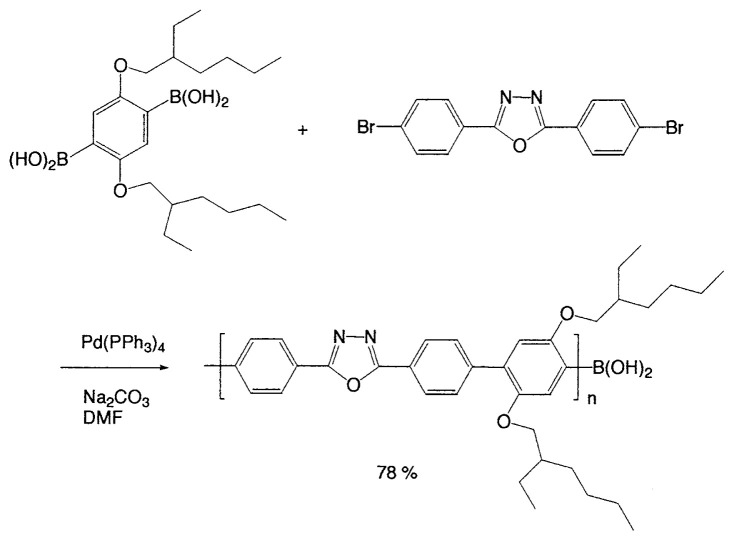


## Coupling reaction of (sp^3^)C-B compounds

Although organometallic reagents with 1-alkenyl, 1- alkynyl, and aryl groups were successfully used for the coupling reactions, those with alkyl groups having (sp^3^)carbons containing ***β***-hydrogens were severely limited due to the competitive side reactions. In 1971–1972 Kochi, Kumada, and Corriu reported independently that the reaction of alkyl Grignard reagents with alkenyl or aryl halides are markedly catalyzed by Fe(III) or Ni(II) complexes, and then Negishi demonstrated the synthetic utility of alkylzinc compounds by use of palladium catalyst. Thereafter, alkyllithium, -tin, and -aluminum reagents were also employed for such cross-coupling reactions.6) The reaction of alkylborane derivatives is particularly useful when one wish to start from alkenes via hydroboration. Consequently, we intended to examine the coupling reactions between alkylboron compounds and various organic halides in the presence of base and palladium complex, and found that no cross-coupling reactions of B-alkyl-9-borabicyclo[ 3.3.1]nonanes (B-R-9-BBN), readily obtainable from alkenes by hydroboration, with 1-halo-1-alkenes or haroarenes occurred under the standard coupling conditions, but the coupling proceeds smoothly by using a catalytic amount of PdCl_2_(dppf) and bases, such as NaOH, K_2_CO_3_, and K_3_PO_4_ to give the corresponding alkenes or arenes in excellent yields (Eq. 21).30) Because the reaction is tolerant of a variety of functionalities on either coupling partner, stereochemically pure functionalized alkenes and arenes can be obtained under mild conditions (Eq. 22). The utility of the reaction was demonstrated by the stereoselective synthesis of 1,5-alkadienes (**26**) (Eq. 23) and the extension of a side-chain in a steroid **27** (Eq. 24).30)

(Eq. 21)
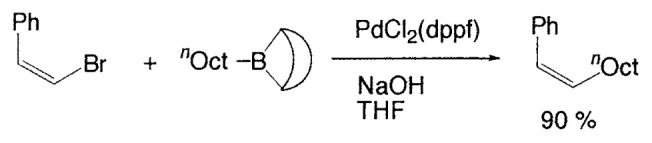


(Eq. 22)
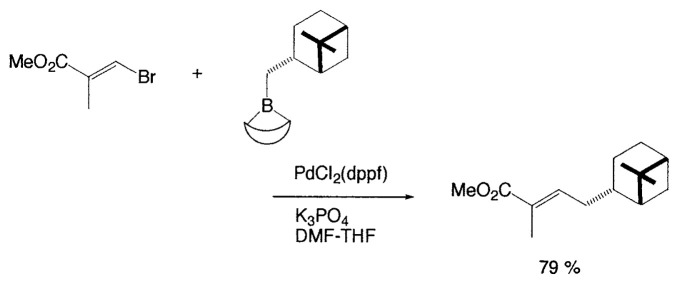


(Eq. 23)
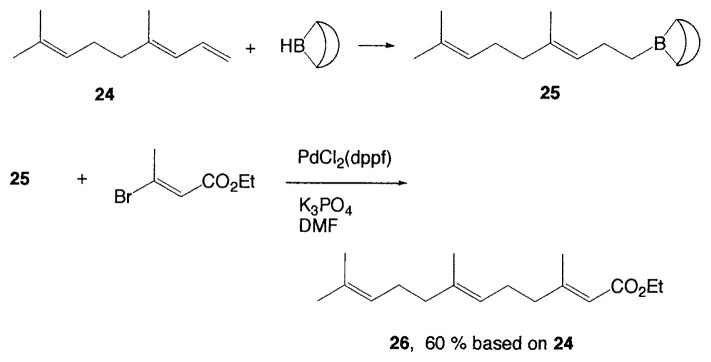


(Eq. 24)
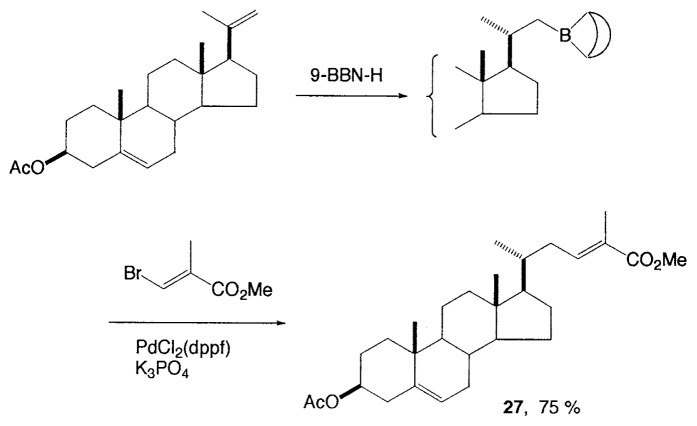


Many chemists applied such a Suzuki coupling reaction using B-saturated alkylboron compounds. For instance, Danishefsky *et al*. reported a total synthesis of the promising anticancer agent (−)-epothilone B using the coupling method as shown below (Eq. 25),31) and a sister compound, epothilone A was also synthesized by the similar procedure.32) The full paper of the total synthesis of epothilones A and B has been appeared more recently.33)

(Eq. 25)
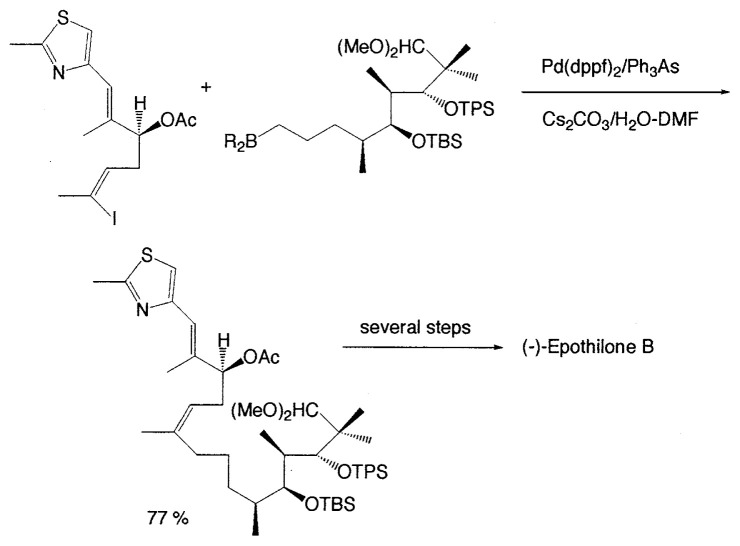


A total synthesis of a potent antitumor agent, desoxyepothilone F has been recently accomplished by Danishefsky *et al*. by using Suzuki coupling as a key reaction. 34)

Marine polyether toxins present challenging synthetic targets due to their structural complexity and exceptionally potent biological activities. The most critical issue in the synthesis of these large polyether compounds is development of synthetic methods for convergent coupling of polyether fragments. In spite of recent advantages in the synthesis of medium-sized cyclic ethers, only a few methodologies for the convergent assembly of 6-membered polyether structure were reported. A new strategy for such synthesis of *trans*fused polyethers based on palladium(0)-catalyzed Suzuki coupling reaction of alkylboranes with cyclic enol triflates has been developed by Tachibana *et al*.35) As shown in Eq. 26, the cross-coupling reaction is carried out in the presence of cesium carbonate as a base and triphenylarsine as a coligand in DMF at room temperature. Further reactions give the expected *trans*-fused polyether.

### Base problem

In cross-coupling reactions of organoboron compounds, the presence of bases is essential; no reaction occurs without base. On the other hand, there are many organic compounds, sensitive toward bases. Consequently, careful uses of bases are required in such cases. For example, Table IX shows that the selection of base and solvent system provides markedly different yields of coupled products. By careful selection of the reaction conditions (e.g., PdCl_2_(dppf)/K_2_CO_3_/DMF), high yields of the desired coupled products can be achieved (Eqs. 27 and 28).

(Eq. 26)
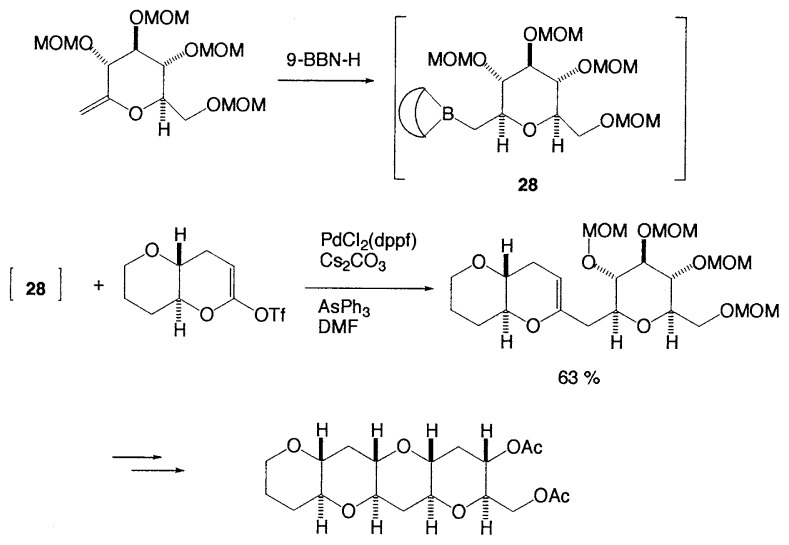


## Coupling reactions of (sp)C-B compounds

Alkynylboranes have long been known to be useful synthetic intermediates. Compared to other organoboranes, they are stronger Lewis acids and are easily hydrolyzed. Because of this property, alkynylboron compounds have not been employed in the Suzuki coupling reaction, in which the presence of bases is essential. Recently, Soderquist *et al*. have found that the addition of B-methoxy-9-borabicyclo[3.3.1]nonane to alkynyllithium reagents gives stable complexes **29** which undergo efficient Suzuki coupling to produce a variety of alkynyl derivatives **30** (Eq. 29, Table X).36)

(Eq. 27)
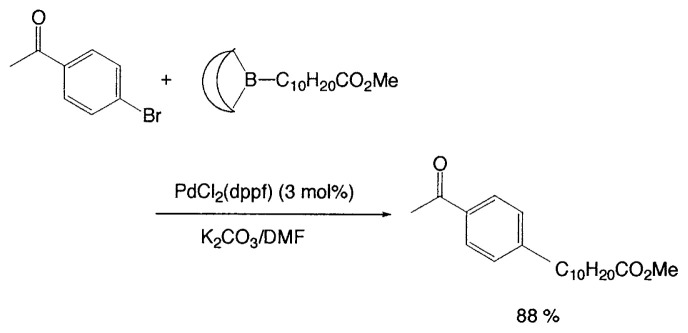


(Eq. 28)
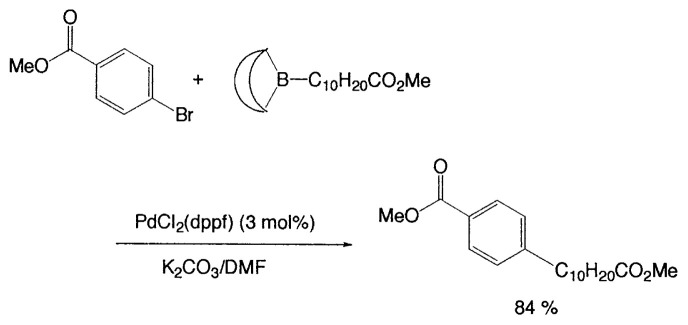


Almost at the same time, Fürstner and Seidel reported the same reaction.37) Namely, the necessary alkynyl borates in the palladium catalyzed C-C bond formation are prepared from 9-methoxy-9-BBN and a polar organometallic reagent RM, such as 1-alkynyl sodium, potassium, and lithium compounds, and not as usually from boranes and bases. This approach allows cross-couplings of organic halides with e.g. alkynyl-, methyl-, or TMSCH_2_-groups. The method is highly chemoselective and turned out to be compatible with aldehyde, amide, ketone, ester and cyano functions as well as with basic nitrogen atoms in the substrates. Some of the results are shown in Table XI. This reaction is used to prepare the compound **31** which is highly valuable for its chemoluminescence property.

(Eq. 29)
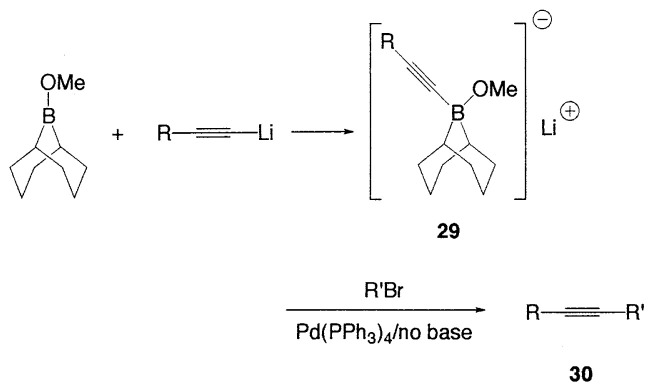


Most recently the palladium-catalyzed cross-coupling reaction of potassium alkynyltrifluoroborates with aryl halides or triflates has been reported to proceed readily to give coupled products. The potassium alkynyltrifluoroborates are air- and moisture-stable crystalline solids that can be stored indefinitely, which will provide an advantage in application to combinatorial chemistry (Eq. 30).38)

(Eq. 30)
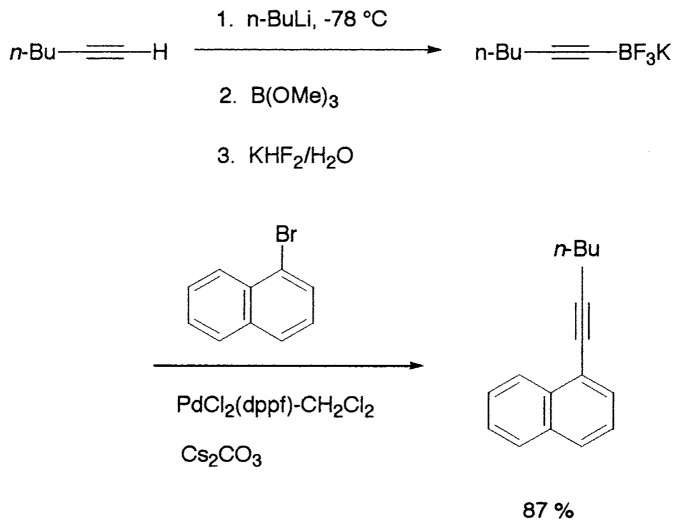


## Conclusion

As shown in this article, all kinds of organoboron compounds including (sp^2^)C-B, (sp^3^)C-B, and (sp)C-B bonds can be used in coupling reactions with various organic electrophiles to provide corresponding coupled products readily in high yields stereo- and regioselectively. Such coupling reactions have a number of advantages which are indicated in the summary.

## Figures and Tables

**Fig. 1:**
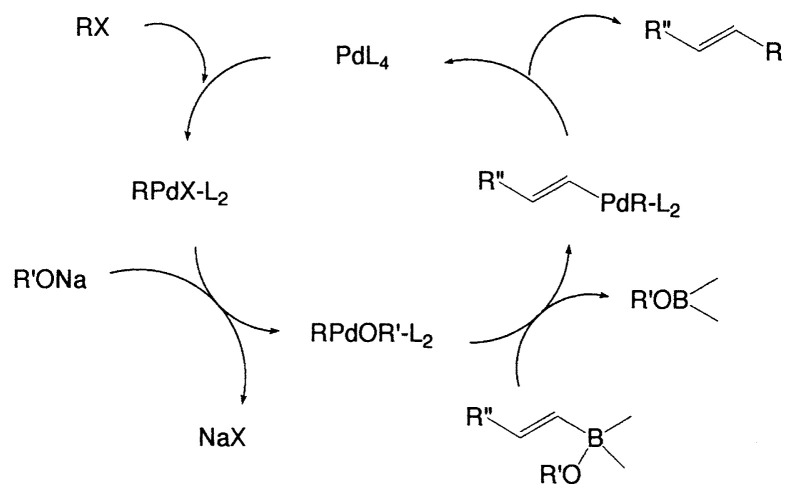
Catalytic cycle for the coupling reaction of alkenylboranes with haloalkenes.

**Table I:** Cross-coupling reaction of **1** with **2**

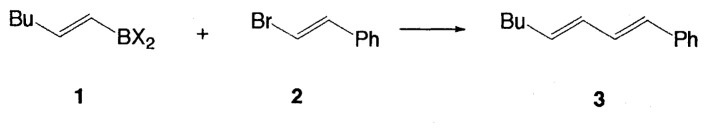					

**1**^a^	Catalyst^b^ (mol%)	Base (Equiv / **2**)	Solvent	Reac. time(h)	Yield (%) of **3**
**1b**	PdL_4_ (3)	None	THF	6	0
**1b**	PdL_4_ (3)	None	Benzene	6	0
**1a**	PdL_4_ (3)	2M NaOEt(2)-EtOH	THF	2	73
**1b**	PdL_4_ (1)	2M NaOEt(2)-EtOH	Benzene	2	86

a) **1a**, X_2_ = (Sia)_2_; **1b, X_2_ =** 
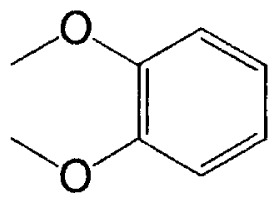

b) L=PPh_3_

**Table II:** Cross-coupling reaction of (E)-1-vinyldisiamylboranes

1-Alkenyl-borane	1-Alkenyl-bromide	Product	Yield/%(Purity/%)
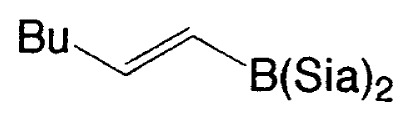	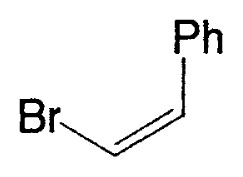	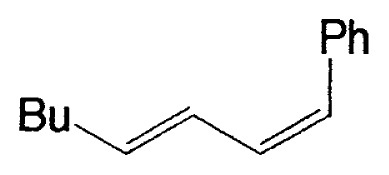	86 (98)
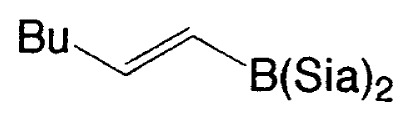	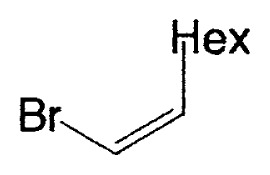	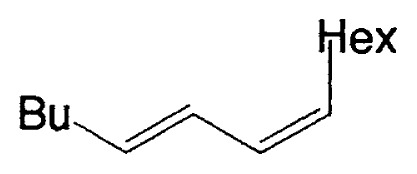	88 (99)
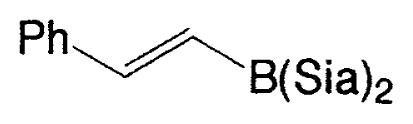	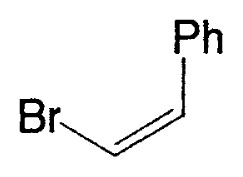	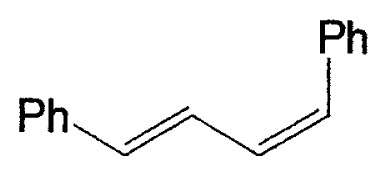	89 (98)

Reaction conditions: Pd(PPh_3_)_4_/NaOEt/benzene/reflux/2 h

**Table III:** Cross-coupling of (Z)-1-hexenyldisiamyl- or (Z)-1-hexenyl-diisopropoxyborane

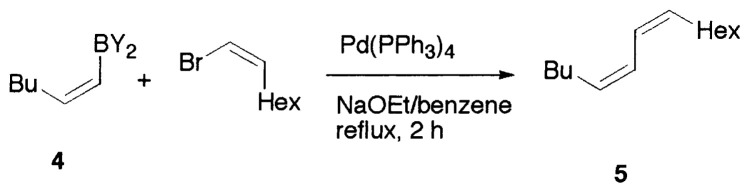		

BY_2_ in **4**	Yield(%) of **5**	Purity(%) of **5**
B(Sia)_2_	49	>98
B(O*^i^*Pr)_2_	87	>99

**Table IV:** Cross-coupling reaction of 10 with iodobenzene

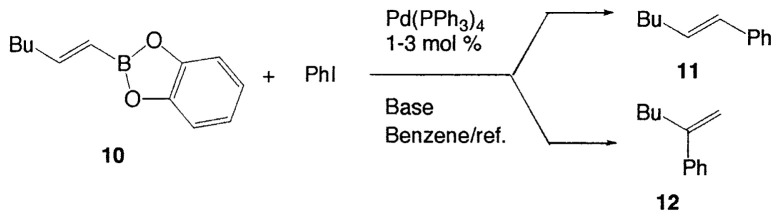			

Base	Reaction time (h)	Product yield (%)	Ratio of **11**:**12**
None	6	0	
NaOEt	2	100	100:0
NaOMe	2	100	100:0
NaOH	2	100	100:0

**Table V:** Coupling of 1-alkenylboranes with various organic halides

1-Alkenylborane	Halide	Product^a^	Yield (%)
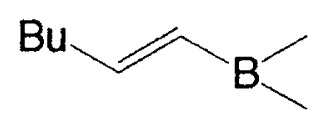	Phl	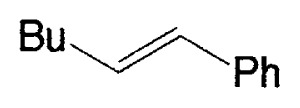	100
	PhBr	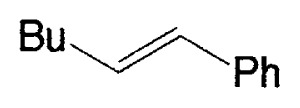	98
	PhCl	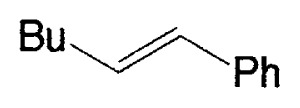	3
	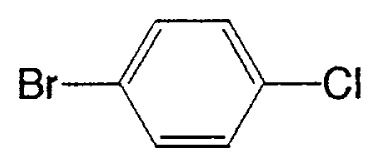	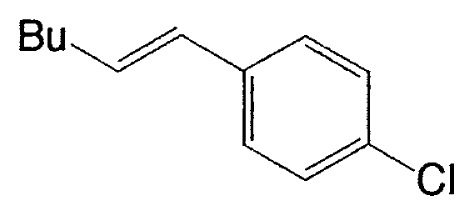	100
	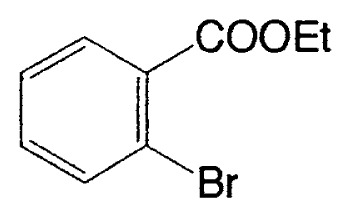	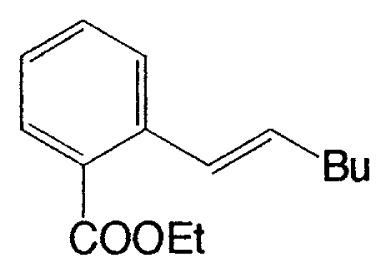	87
	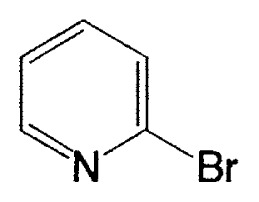	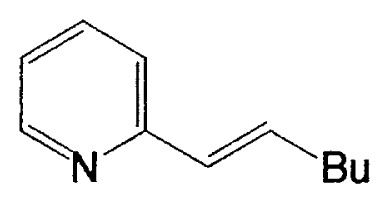	83
	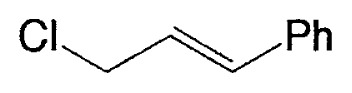	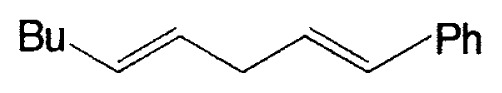	89
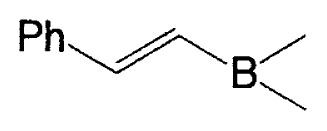	PhCH_2_Br	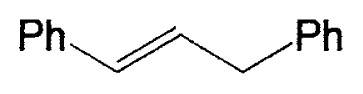	97
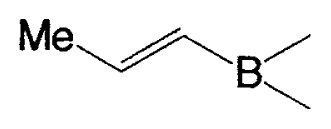	BrC≡CPh	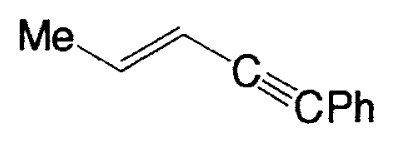	93
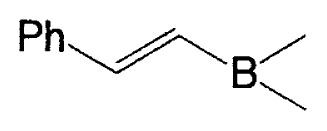	BrC≡CHex	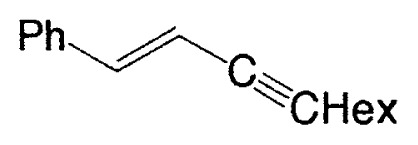	95

a) Isomeric purity, >98 %

**Table VI:** Reaction of mesitylboronic acid with iodobenzene under different conditions

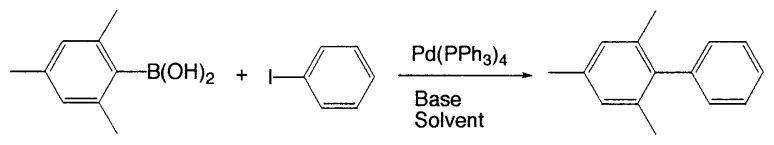					

Base	Solvent	Temp/°C	Yield/%^a^		
			Time 8 h	24 h	48 h
Na_2_CO_3_	Benzene/H_2_O	80	25(6)	77(12)	84(25)
Na_2_CO_3_	DME/H_2_O	80	50(1)	66(2)	83(7)
K_3_PO_4_	DME/H_2_O	80	70(0)		
NaOH	DME/H_2_O	80	95(2)		
Ba(OH)_2_	DME/H_2_O	80	99(2)		

a GLC yields of the coupling product based on iodobenzene and the yields of mesitylene are shown in the parentheses.

**Table VII:** Ligand effects in the coupling of hindered substrates

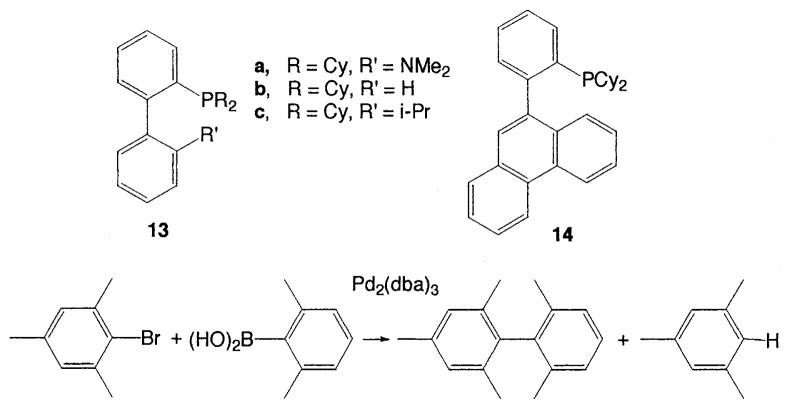			

ligand	conv (%)	biaryl (%)	biaryl/Ar-H
**13a**	47	33	2.3
**13b**	20	10	0.9
**13c**	74	40	1.9
**14**	100	91	10

**Table VIII:** Suzuki couplings of unactivated aryl chlorides

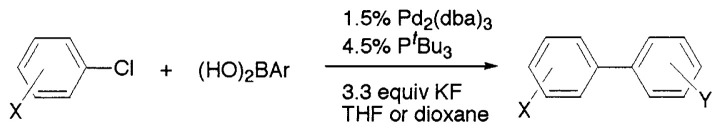				

Aryl Chloride	Boronic Acid	Product	Temp(°C)	Yield (%)
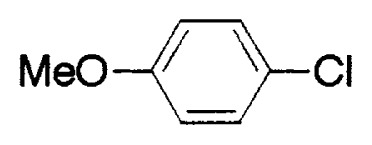	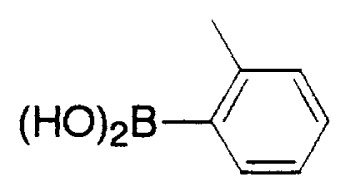	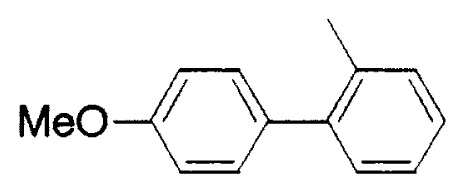	70	88
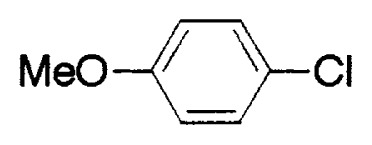	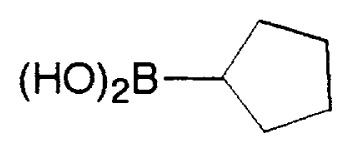	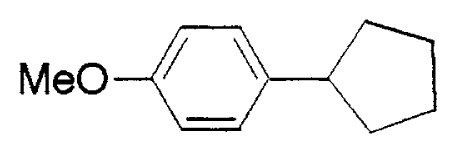	100	75

**Table IX:** Solvent and base effects on the cross-coupling reactiona)

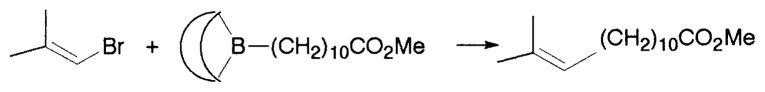				

Solvent	Base (equiv)	Temp.(°C)	Time (h)	Yield (%)
DMF	KOAc (4)	50	18	18
DMF	K_2_CO_3_ (2)	50	18	64
CH_3_CN	K_2_CO_3_ (4)	50	18	46
DMF	K_3_PO_4_ (4)	50	20	92

a) Catalyst: PdCl_2_(dppf)

**Table X:** Coupled products from **29**

R	R’	Product yield(%)^a^
*n*-Bu	C_6_H_5_	60 (92)
SiMe_3_	C_6_H_5_	64
Ph	C_6_H_5_	64
*n*-Bu	*p*-MeOC_6_H_4_	62 (68)
SiMe_3_	CH_2_=CC_6_H_5_	88
*t*-Bu	*cis*-CH=CH-t-Bu	56
SiMe_3_	*trans*-CH=CH-n-Bu	55

a) Isolated yields of analytically pure compounds (GC yields)

**Table XI:** Pd-catalyzed arylation of alkynyl metal reagents mediated by 9-MeO-9-BBN derivatives

Substrate	RM	Product	Yield/%
4-bromobenzophenone	MeC=CNa	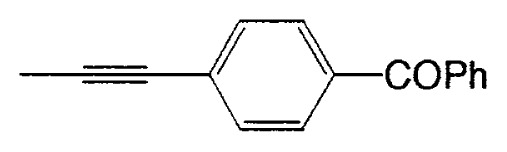	89
4-bromobenzaldehyde	PhC=CK	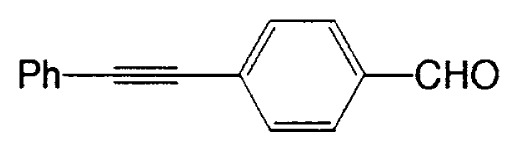	77
ethyl 4-bromobezoate	MeC=CNa	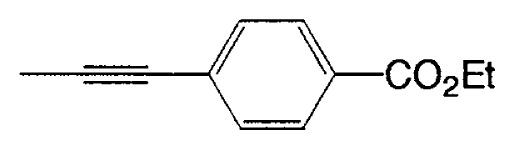	86
4-bromobenzonitrile	PhC=CK	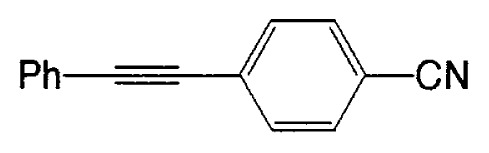	93
9,10-dibromoanthracene	PhC=CLi	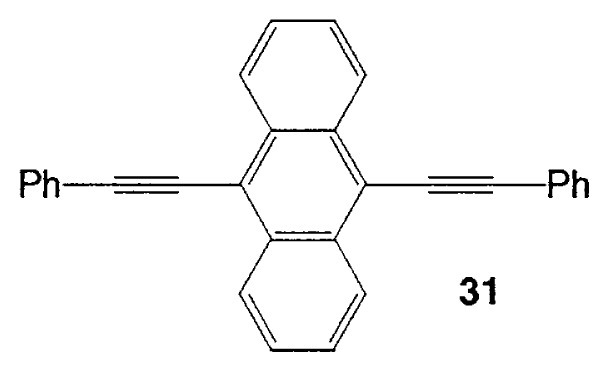	84
